# Effects of *Thymus hirtus sp. algeriensis* Boiss. et Reut. (Lamiaceae) essential oil on healing gastric ulcers according to sex

**DOI:** 10.1186/1476-511X-13-138

**Published:** 2014-08-26

**Authors:** Fatma Guesmi, Manel Ben Ali, Taha Barkaoui, Wiem Tahri, Mondher Mejri, Mossadok Ben-Attia, Houda Bellamine, Ahmed Landoulsi

**Affiliations:** Laboratory of Biochemistry and Molecular Biology, Faculty of Science of Bizerte, Bizerte, Tunisia; Laboratory of Biomonitoring of the Environment (LR01/ES14), Faculty of Sciences of Bizerte, Bizerte, Tunisia; Higher Institute of Technological Studies (ISET), Mogran, Zaghouan, Tunisia; Service of Anatomo- Pathology of Menzel Bourguiba, Bizerte, Tunisia

**Keywords:** *Thymus algeriensis*, Gastric ulcers, HCl/ethanol, Antioxidant enzymes

## Abstract

**Background:**

*Thymus algeriensis* Boiss. et Reut. (Lamiaceae), popularly known as “mougecha” or “mazoukcha” is prolific in Mediterranean regions, mostly in North Africa, and is used in folk medicine to treat of stomach diseases.

**Methods:**

In this study, animals were induced with gastric ulcers using HCl/ethanol (0.3 M HCl/60% ethanol) and treated orally with essential oil of *Thymus algeriensis* (EOTa) in various doses ranging from 54 mg/kg body weight to 180 mg/kg body weight.

**Result:**

The dose found to be effective was 180 mg/kg body weight, since this dose brought about a maximum reduction in lesion index in female rats. In gastric tissues, levels of total glutathiones (GSH, GST and GPx) and thiobarbituric acid reactive substances (TBARS) were evaluated. The activities of the antioxidant enzymes, catalase (CAT) and superoxide dismutase (SOD) were measured. Histopathological changes were observed using a cross section of gastric tissue. Chemical analysis revealed the presence of 13 components accounting for 77.7% of the essential oil from dried leaves. Oral administration of EOTa (54, 117 and 180 ml/kg) inhibited HCl/ethanol-induced ulcers. Lesion index was significantly reduced in ulcer induced animals treated with EOTa (HCl/ethanol + EOTa) compared to those ulcerated with HCl/ethanol but with no treatment given. Females showed a greater resistance to ulcers and gastric lesions occurred less often than in males. GSH, pH, enzymic antioxidants, and adherent mucus content were all significantly increased.

**Conclusion:**

From the data presented in this study, it can be concluded that male rats are more sensitive to gastric ulcers induced by HCl/ethanol than females.

## Background

A peptic ulcer is an excoriated area of the gastric or duodenal mucosa caused by gastric juice action. It is both a chronic and recurrent disease, and the most predominant of the gastrointestinal diseases [1]. Potentially injurious agents such as acid, pepsin, bile acids, food ingredients, bacterial products and drugs have been implicated in the pathogenesis of gastric ulcers, including increased gastric acid and pepsin secretion, decreased gastric blood flow, suppression of endogenous generation of prostaglandins, inhibition of mucosal growth and alteration of gastric mobility [2]. Some evidence suggests that reactive oxygen species play a role in the etiology and pathogenesis of digestive system disorders such as gastrointestinal inflammation and gastric ulcers [3].

ROS could either directly disrupt the mitochondrial membrane that subsequently leads to the release of cytochrome C which becomes a part of the apoptosome complex, or it could lead to membrane rupture of lysosomes in an additive way resulting in the release of cathepsins which activate caspase and an apoptosis cascade, finally leading to cell death *via* apoptosis. On the contrary, cells protect themselves against the destructive effects of ROS by scavenging them through the enzyme defense system, or through the antioxidant activities of dietary compounds [4]. After exposure to oxidative stress, the defense mechanisms including an enzymatic system such as superoxide dismutase (SOD), catalase (CAT), glutathione peroxidase (GPx) and non-enzymatic system such as glutathione (GSH), β-carotene (vitamin A), ascorbic acid (vitamin C) and α-tocopherol (vitamin E) are involved [5, 6].

The stomach can activate many gastroprotective mechanisms to prevent injury from noxious agents. Of these defensive factors, several studies have recently demonstrated that the gastric mucus offers protection [7, 8]. When it is overwhelmed or breaks down due to disease, the second line of defense includes intracellular acid neutralization, rapid epithelial repair and maintenance, and redistribution of gastric vasculature [9].

The current medicinal treatment of peptic ulcers is generally based on inhibition of gastric acid secretion by H2- blockers, omeprazole and antimuscarinics, as well as on acid-independent therapy provided by sucralfate and bismuth. The first proton pump inhibitor (PPI) used clinically was omeprazole (2-[[3,5-dimethyl-4-methoxypyridin-2- yl]methylsul®nyl]-5-methoxy-1H-benzimidazole). Compounds in this class are acid-activated prodrugs [10]. In cases of *Helicobacter pylori* infection, antibiotics are used. Obviously, drugs endowed with antisecretory activity coupled with gastroprotective effects could be a promising approach for successful treatment of peptic gastric ulcers because of the potential complementary effects of therapeutic modalities acting via different mechanisms [11].

It has been suggested that experimental ulcers induced by ethanol administration cause more severe gastric erosion in male rats than in females [12]. The female sex has a greater resistance to stress and thus, gastrointestinal lesions happen less often than in the male sex [13]. Males have a well-known risk of stroke and, in most epidemiological series, stroke occurs more frequently in men than women. This sexually dimorphic disease pattern remains apparent until an age well beyond the menopausal years [14]. The development of new antiulcer drugs and the search for novel molecules has therefore been extended to herbal drugs that offer better protection and decreased relapse. Medicinal plants provide an effective and safer way to manage disease. Many medicinal plants exhibit antiulcer activity and were found useful in the treatment of peptic ulcers [15].

Generally, antiulcerogenic compounds obtained from plants exert their effects either by stimulating the protective factors of gastric mucosa due to increased synthesis of prostaglandins, by stimulating the secretion of mucus and bicarbonate through inhibition of acid secretion by interacting with different receptors, or by regulating enzymes or hormones involved in the secretory process [16].

Herbal medicines have now triumphed as a diverse popular therapy and are emerging as an alternative treatment to the synthetic drugs available [17, 18]. The latest trends have shown an increasing demand for phytodrugs and some medicinal herbs have been proven to have antiulcer activity. This alone is an important reason to investigate the antiulcer effects of medicinal plants through traditional use in preventing gastric disease. Essential oils are an important part of traditional pharmacopoeia in many countries and have been successfully used for gastroprotection and healing ulcers. Essential oils are complex mixtures made up of many single compounds chemically derived from terpenes and their oxygenated compounds. These constituents contribute to the antifungal, antiviral and antioxidant effects [19, 20]. Of those plants known for their medicinal value, the plants of the genus thymus belonging to the Lamiaceae family (common name: Mougecha or Mazoukcha) are very important for their therapeutic potential. *Thymus algeriensis*, which is commonly found throughout North Africa, has been widely used in traditional medicine as an antiseptic and antispasmodic. Furthermore, this plant also has widespread use in folk medicine against illnesses of the digestive tube and for antiabortion [21]. Recently, *T. algeriensis* essential oil was found to possess an interesting inhibitory activity towards the angiotensin I-converting enzyme suggesting the potential of this plant as an antihypertensive agent [22].

Thus, the present study intends to explore the ulceroprotective and antioxidant activity of the essential oil of this species on HCl/ethanol- induced ulcers in rats. Our aim was to investigate whether sex had an influence on healing changes in the gastric acid secretion and blood flow at the margin of the ulcer.

## Material and methods

### Plant material

The aerial part of *Thymus sp. algeriensis* was collected on Mount Orbata (Jbel Orbata) near Zannouch, Gafsa, Tunisia. No specific permission was required to take plants from these locations and the field studies did not involve endangered or protected species. The plant material was authenticated by Mr. Hamdi Lazhar, Engineer and Director of Bouhedma Natural Park and the voucher specimens were deposited in the Herbarium of the National Institute of Agronomy of Tunisia (INAT) for future reference.

### Extraction and chromatographic analysis of the essential oil of *Thymus algeriensis*(EOTa)

The essential oil from dried powdered *Thymus algeriensis* aerial parts was isolated by steam distillation in a Clevenger-type apparatus according to Procedure III of the Yugoslav Pharmacopoeia IV [23]. Essential oil yield was 2.3% (w/w). Freshly isolated essential oil was a yellow liquid with an intense, necrotic odor.

Samples of 1 μl (dilution in hexane 10%) were subjected to analysis by GC-MS. GC analysis was performed on a model 7890 A (series II) gas chromatograph, with a flame ionization detector (FID) and a split ratio of 1:50 using a fused silica capillary column, HP5-MS (30 m × 250 μm i.d., 0.25 μm film thickness). Injector or detector temperature for each analysis was about 250°C, and the carrier gas was helium with a flow rate of 0.8 ml/min. Peak areas were measured by electronic integration, and relative amounts of the individual components were based on the peak areas. GC-MS was carried out on an Agilent model 5975 C mass spectrometer operating at ionizing energy mode at 70 eV, combined with the GC described above.

### Preparation of drug solution

One hundred grams of the air dried AERIAL PART OF *Thymus algeriensis* were steam distilled for 6 h to prepare the appropriate stock solution of the drug, i.e. 54 mg/ml, 117 mg/ml and 180 mg/ml. Complete analyses of the samples are in progress. For the pharmacological tests carried out here, the complete essential oil was emulsified in vehicle (0.1% Tween 80 aqueous solution) before administering it to the animals. The doses were administered orally by selecting the appropriate concentration of the stock solution. Omeparazole was dissolved in vehicle and given orally to the reference control group (6 rats) in doses of 20 mg/kg body weight.

### Experimental protocol

A total of 54 Adult male and female Wistar rats (weighing 150 to 180 g and housed six per cage) were obtained from the Animal Laboratory of the Pasteur Institute of Tunis, Tunisia (Ethics No. LNSP/Pro 152012). They were provided with food and water *ad libitum* and were housed in polypropylene cages under pathogen free, uniform conditions of light and dark cycles (12 h each). Temperature was kept constant at 25°C ± 2°C. Rats were randomly divided into 9 groups (n = 6/group) and made to fast for 24 h with free access to water prior to the experiment. Group 1 (Female rats) and Group 2 (Male rats): the animals were given an oral dose of 0.5 ml of vehicle (0.1% tween-80 aqueous solution) (Table 1); 80 mg/ml of a solution containing 0.3 M HCl/60% ethanol (HCl/ethanol) to induce gastric ulcers was administered overnight to fasting rats. Group 3: (Standard drug treated group): rats were treated with omeprazole (20 mg/kg/p.o) one hour before they were subjected to the HCl/ethanol treatment. Groups 4, 5, and 6 received EOTa (54, 117 and 180 ml/kg, p.o treated female rats) and Groups 7, 8 and 9 received EOTa (54, 117 and 180 ml/kg, p.o treated male rats) treated with essential oil of *T.algeriensis* dissolved in a 0.1% tween 80% aqueous solution. HCl/ethanol (80 mg/ml) was administered after one hour to induce ulcers. Animals were sacrificed 1 h after administering HCl/ethanol and the stomachs were excised and inflated by saline injection (2 ml) to determine the ulcer index. All rats were sacrificed under ether anesthesia, and all efforts were made to minimize suffering. Throughout the experiments, all animals were treated humanely. All experiments were performed in the morning and in accordance with the guidelines provided by the Institutional Animal Ethics Committee. The gastroprotective effect of EOTa was assessed from lipid peroxide (LPO), reduced glutathione (GSH), and activities of enzymic antioxidants—super oxide dismutase (SOD), catalase (CAT), glutathione peroxidase (GPx), and glutathione-*S*-transferase (GST) in gastric mucosa.

**Table 1:** **The experimental design and specifications**

Groups number	Description	Pre-treatment	Treatment
Group 1	Control (female rats)	0.5 ml of vehicle (0.1% tween-80 aqueous solution)	(80 mg/ml) HCl/ethanol
Group 2	Control (male rats)	0.5 ml of vehicle (0.1% tween-80 aqueous solution)	(80 mg/ml) HCl/ethanol
Group 3	Reference control	Omeprazole 20 mg/kg	(80 mg/ml) HCl/ethanol
Group 4	Experimental group 1 (male rats)	Complex 54 mg/kg	(80 mg/ml) HCl/ethanol
Group 5	Experimental group 2 (male rats)	Complex 117 mg/kg	(80 mg/ml) HCl/ethanol
Group 6	Experimental group 3 (male rats)	Complex 180 mg/kg	(80 mg/ml) HCl/ethanol
Group 7	Experimental group 4 (female rats)	Complex 54 mg/kg	(80 mg/ml)HCl/ethanol
Group 8	Experimental group 5 (female rats)	Complex 117 mg/kg	(80 mg/ml)HCl/ethanol
Group 9	Experimental group 6 (female rats)	Complex 180 mg/kg	(80 mg/ml)HCl/ethanol

### Macroscopic gastric lesion evaluation

After washing with normal saline, gastric lesions were quantified and ulcers were scored according to the method used by Dashputre and Naikwade [24]. The ulcer scores were as follows:

.0: normal colored stomach.

.0.5: red coloration.

.1: spot ulcers.

.1.5: haemorrhagic streak.

.2: deep ulcers.

.3: perforation.

The mean ulcer score for each animal was expressed as an ulcer index. The percentage of ulcer protection was determined as follows:

The ulcer index score for each animal was expressed as an ulcer index. The percentage of ulcer protection was determined as follows:

The ulcer index (UI) was measured using following formula:

UI = UN + US + UP × 10^− 1^ Where, UI = Ulcer Index; UN = Average number of ulcers per animal;

US = Average severity score; UP = Percentage of animals with ulcers.

The mean ulcer score for each animal was expressed as an ulcer index. The percentage of ulcer inhibition was determined as follows:


### Histology of gastric lesions

Samples of gastric tissue were fixed in 10% buffered formalin. The stomach was sectioned at 5 μm and stained with Hematoxylin Eosin for histological assessment.

### Assessment of oxidative stress in tissue

In order to determine the effect of EOTa on oxidative stress induced in the HCl/ethanol model, the levels of GSH, TBARS and activities of SOD, CAT, GPx and GST were measured in gastric tissue.

### Preparation of homogenate

The stomachs were weighed and homogenized in a buffer solution of potassium phosphate (pH 7.4) and centrifuged at 3,000 rpm/15 min. The supernatant was used for the enzymatic and MDA assays.

### Estimation of protein

Protein content of the gastric tissue was determined by the Folin Lowry Method using a bovine serum albumin as a standard [25].

### Determination of total glutathione (GSH)

Reduced glutathione was estimated by the method indicated by Sedlak and Lindsay [26]. The homogenate was immediately precipitated with 0.1 ml of 25% trichloroacetic acid, and the precipitate was removed by centrifugation at 4200 rpm for 40 min at 4°C. The precipitated tissue homogenate was treated with 5,5′-dithiobis(2- nitrobenzoic acid) (DTNB) reagent. A standard calibration curve was prepared using reduced glutathione (GSH). Absorbance was measured at 412 nm using a spectrophotometer. The amount of glutathione was expressed as μmol/mg protein.

### Estimation of SOD antioxidant enzyme

Enzyme activity was assayed by following the inhibition of pyrogallol auto-oxidation [27]. Pyrogallol (24 mmol/L) was prepared in 10 mmol/L HCl and stored at 4°C. CAT 30 μmol/L stock solution was made in an alkaline buffer (pH 9.0). Aliquots of supernatant were added to a Tris–HCl buffer containing 12.5 μl of pyrogallol and 12.5 μL of CAT stock solutions. The total reaction mixture was made to 1.425 ml with the same Tris–HCl buffer. Auto-oxidation of pyrogallol was monitored by measuring absorbance at 420 nm at 1-minute intervals for 5 minutes. SOD activity was determined from a standard curve of percentage of inhibition of pyrogallol auto-oxidation with a known SOD activity. One unit of SOD is defined as the amount that shows 50% inhibition at room temperature and a pH of 7.8.

### Estimation of catalase (CAT) activity

5 ml solution of 0.01 M hydrogen peroxide was prepared with the buffer solution and used as a substrate for the assay. 20 μl of the supernatant sample was mixed with 780 μl of buffer/hydrogen peroxide and 200 μl of distilled water. The enzyme activity was measured at 240 nm by spectrophotometry for 60 s by reading the change in absorbance between the fifteenth and sixtieth second. The results are expressed in μmol H_2_O_2_/min/mg of protein [28].

### Estimation of GST activity

GST was assayed with the method used by Habig [29] by adding 1-chloro-2,4-dinitrobenzene (CDNB). Change in optic density was read at 340 nm for 3 min at an interval of 30 s. The GST activity was expressed as nmol of CDNB conjugate formed/min/mg protein.

### Estimation of GPx activity

GPx activity was measured using a modified version of that used by Hafeman [30]. The reaction mixture contained GSH, phosphate buffer, H_2_O_2_, TCA (trichloroacetic acid), Na_2_HPO_4_ and DTNB (5, 5^′^-dithiobis (2-nitrobenzoic acid)). The rate of reaction was measured by the decrease in GSH, which was determined by measuring the reaction products of DTNB and GSH (absorbance of the ions at 412 nm). One unit of enzyme activity was defined as a decrease of 1 μmol/L of GSH concentration at 37°C and pH 6.5, while non-enzymatic reactions were excluded.

### Estimation of lipid peroxide

Lipid peroxide content in gastric mucosal tissues was determined by thiobarbituric acid reaction as described by Ohkawa [31]. The lipid peroxide concentration was expressed as nmol MDA/mg protein.

### Measurement of mucus production

The gastric mucosa of each rodent was gently scraped using a glass slide and the mucus obtained was weighed using a precision electronic balance [32].

### Acute toxicity study in rodents

Adult male and female Wistar Dawley rats (6–8 weeks old; 150–180 g) were obtained from the Experimental Animal House, Pasteur Institute, University of Tunisia. The rodents were given standard pellets as food and clean tap water. Thirty six rats were assigned to six groups of six rats each. The female rats were divided into control, low dose and high dose groups and the same approach was taken for the males. The rodents fasted overnight, then were given doses of EOTa at 300 and 500 mg/kg body weight, and continued fasting for 3–4 h after dosing every day for 14 days. Behavioral changes, weight, consumption of food and water, clinical signs of toxicity, and mortality were recorded daily [33].

### Statistical analysis

The values were reported as mean ± S.E.M. The significance (p < 0.05) of the results was assessed by one-way analysis of variance (ANOVA), followed by Bonferroni’s test for multiple comparison or Dunnett’s multiple range test.

## Results and discussion

**Figure 1:**
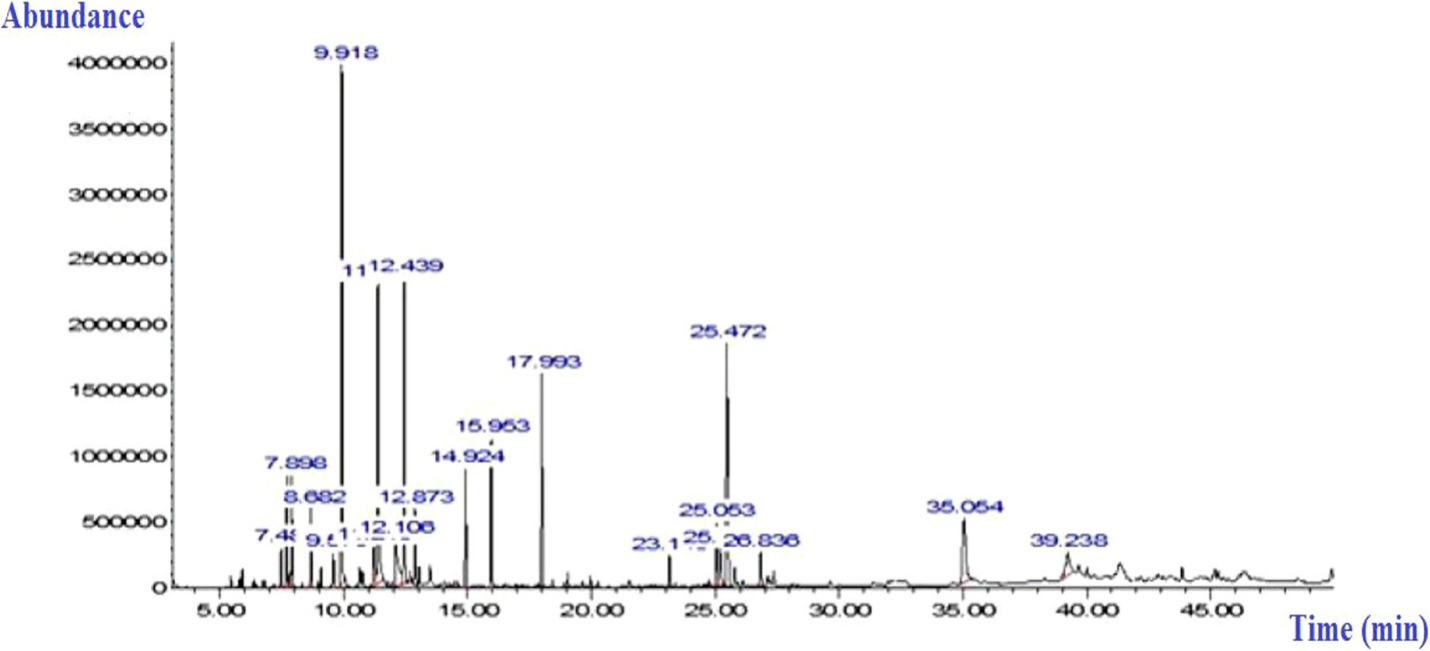
**Chromatographic profile of EOTa using gas chromatography coupled to a mass spectrometer.**

In this investigation, GC/MS analysis made it possible to identify 13 compounds in EOTa, which accounting for 77.75% of the total oil content. The chromatogram in Figure 1 shows four main components in EOTa (Table 2): Linalool (18.05%), Camphor (13.03%), 4-carvomenthenol (11.2%) and Viridiflorol (11.71%). Extraction procedures for EOTa resulted in a yield of 2.36%. Previous chemical reports of the essential oil obtained from 14 Tunisian natural populations of *Thymus algeriensis* leaves collected from different geographical regions revealed that the major components were 1,8-cineole (17.7%), α-pinene (15.5%), and camphor (8.2%) [34]. Zouari *et al*. [35] found that the major compounds of 8 *T. algeriensis* populations analyzed were α-pinene (7.41-13.94%), 1,8-cineole (7.55-22.07%), *cis*-sabinene hydrate (0.10-12.95%), camphor (1.55-11.86%), terpenyl acetate (0–14.92%) and viridiflorol (0–11.49%). In terms of its phytochemical constituents, *Thymus algeriensis* generally contains major classes of secondary metabolites such as flavonoids, phenolic compounds and triterpenoids, to name but a few. The presence of secondary products in fairly high concentrations has commonly been used to explain the claimed curative and palliative efficacy of a variety of traditional herbal medicines and the reports of profound beneficial effects of certain foodstuffs on health [36]. This finding contributed to the antiulcer pharmacological validation of this species, lending more credence to clinical applications for the traditional treatment of stomach complaints symptomatic of peptic ulcer disease.

**Table 2:** **Chemical composition of essential oils extracted from** ***Thymus algeriensis*** **using analysis by GC-MS**

NO.	RT^a^	RI^b^	Components^c^	Peak area (%) EOTa^d^	Identification methods^e^
1	7.481	1017	α –terpinene	1.18	GC-MS-RI^f^
2	7.705	1025	ρ-cymene	3.22	GC-MS
3	7.899	1031	1,8-cineole	3.45	GC-MS
4	8.683	1060	γ-terpinene	2.43	GC-MS-CAS^g^#
5	9.576	1089	Terpinolene	1.35	GC-MS-CAS#
6	9.919	1098	Linalool	18.05	GC-MS
7	11.378	1146	Camphor	13.03	GC-MS
8	12.431	1176	4-carvomenthenol	11.2	GC-MS
9	15.956	1289	Bornyl acetate	5.41	GC-MS
10	23.142	1511	γ-cadinene	1.21	GC-MS
11	25.054	1578	Spathulenol	2.80	GC-MS
12	25.197	1582	Caryophyllene oxide	2.09	GC-MS
13	25.471	1590	Viridiflorol	11.71	GC-MS

^a^RT (retention time), ^b^RI (retention index) measured relative to *n*-alkanes (C_10_ –C_15_); ^c^Components listed in order of their retention index on HP-5 column, ^d^Plant codes; ^e^GC-MS: identification based on a high match of mass spectra retention; ^f^RI Identified by retention index and compared with those reported in the literature [37]; ^g^CAS # = Chemical Abstracts service reference number compared with those reported in the literature [38].

The equilibrium between the therapeutic versus toxicological effects of a drug is a vital parameter in assessing its applicability in relation to pharmacological action [39]. As a part of this pharmacological study, EOTa was investigated for its acute and general toxicity in rodents. In the acute toxicity study, EOTa at doses of 300 and 500 mg kg^−1^ exhibited no signs of toxicity. Anatomical results showed an absence of abnormal organic damage to the rats’ organs.

**Figure 2:**
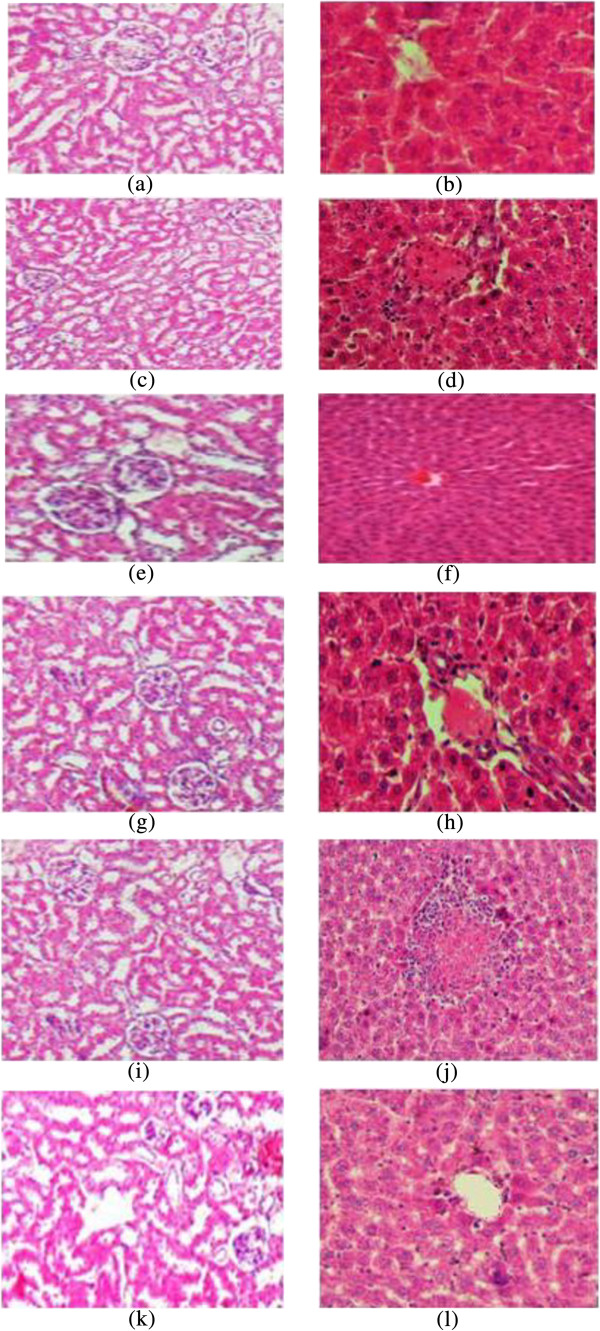
**Histological sections of liver and kidney from the acute toxicity test.** Female rats (1**a** and 1**b**): Control group; female rats (1**c** and 1**d**): treated with 300 mg/kg of EOTa; female rats (1**e** and 1**f**) treated with 500 mg/kg of EOTa; male rats (1**g** and 1**h**): control group; male rats (1**i** and 1**j**): treated with 300 mg/kg of EOTa; male rats (1**k** and 1**l**) treated with 500 mg/kg of EOTa. There is no significant differences in the structure of liver and kidney between treated and control groups (Hematoxylin & Eosin stain 20 × magnifications).

**Figure 3:**
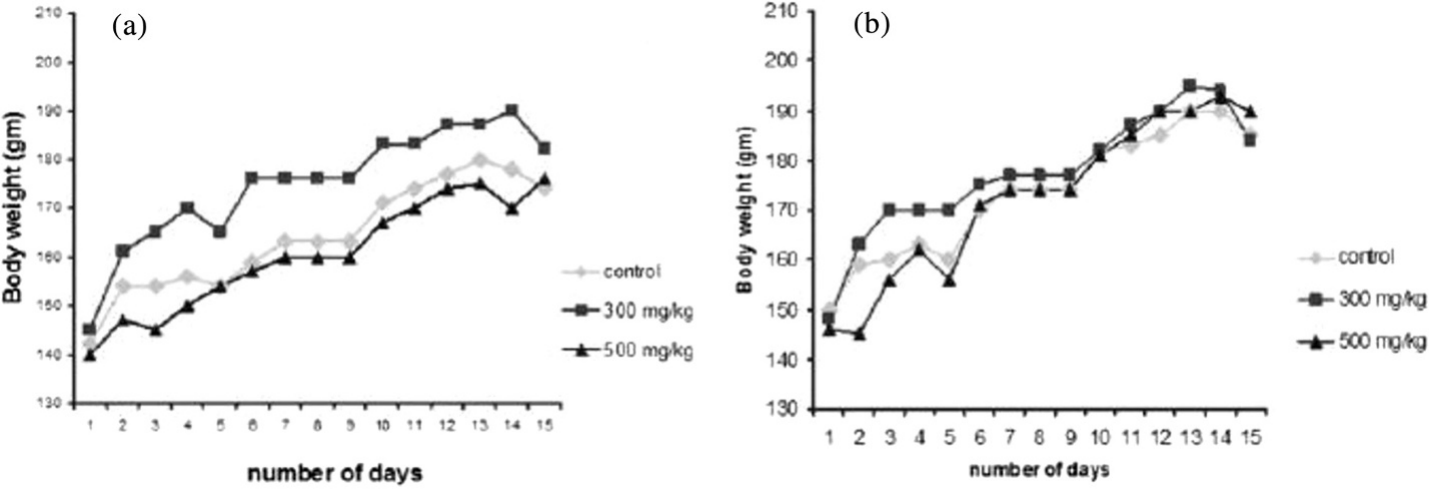
**Body weight gain in rats treated orally with vehicle, Thyme (EOTa, 300 mg/kg), and Thyme (EOTa, 500 mg/kg) for 15 days.** Results are mean ± SEM, n = 6. ANOVA, P > 0.05 between groups in the same day. **(a)** for male rats and **(b)** for female rats.

Neither female nor male rats experienced any toxicity or mortality and there were no abnormal physiological or behavioral changes, nor alterations in body weight at any time during the 14 days of observation (Figure 2 and Figure 3). Histological examination of the liver and kidney did not show any difference compared to the control group.

EOTa treatment (54–180 mg/kg, p.o.) caused a dose-dependent reduction in HCl/ethanol-induced gastric lesions, decreasing the ulcer index and percentage of inhibition mainly at doses of 180 mg/kg for male rats (88%) and between 117 and 180 mg/kg for female rats (96.25 and 98.85%) (Table 3). In this study, omeprazole can protect the gastric mucosa against HCl/ethanol-induced ulceration (65.95%).

**Table 3:** **Measurement of the total no. of ulcers, ulcer index, inhibition percentage, mucus and pH**

Groups number	Treatment (p.o)	PH	Mucus production (μg/g wet tissue)	Total no. of ulcers (mean ± SD) (n = 6)	Ulcer index (mean ± SD) (n = 6)	% ulcer inhibition
Group 1	Control (FR^a^)	2.7 ± 0.65^*^	0.85 ± 0.52^*^	14 ± 2.20^*^	17 ± 1.80^*^	0.00^*^
Group 2	control (MR^b^)	2.95 ± 0.21^*^	0.43 ± 0.14^*^	16.5 ± 4.20^*^	23.5 ± 7.30^*^	0.00^*^
Group 3	Omeprazole	4.37 ± 0.32^**#**^	1.2 ± 0.20^**#**^	5 ± 2.10^**#**^	8 ± 6.13^**#**^	65.95^**#**^
Group 4	EOTa (MR)	3.1 ± 0.80^*#^	0.42 ± 0.40^*#^	13 ± 0.70^*#^	14.83 ± 1.70^*#^	36.90^*#^
Group 5	EOTa (MR)	3.42 ± 0.87^*#^	0.8 ± 0.20^*#^	10 ± 1.80^*#^	11.42 ± 3.80^*#^	51.40^*#^
Group 6	EOTa (MR)	5.79 ± 0.22^*#^	2.2 ± 0.80^*#^	1.5 ± 0.32^*#^	2.82 ± 2.21^*#^	88.00^*#^
Group 7	EOTa (FR)	5.13 ± 0.26^*#^	2.55 ± 0.32^*#^	3.5 ± 2.70^*#^	5.3 ± 1.67^*#^	77.44^*#^
Group 8	EOTa (FR)	5.88 ± 0.20^*#^	2.85 ± 0.46^*#^	0.84 ± 1.33^*#^	0.88 ± 0.22^*#^	96.25^*#^
Group 9	EOTa (FR)	6.14 ± 0.38^*#^	3.18 ± 0.72^*#^	0.25 ± 0.05^*#^	0.27 ± 0.93^*#^	98.85^*#^

^a^FR: Female Rats; ^b^MR: Male Rats; All values are expressed as mean 6 standard error mean. Mean difference is significant at the p < 0.05 level (ANOVA followed by Dunnett’s test). ^*^significant when compared to the ulcer control groups (a and b). ^#^significant when compared to the reference control group (c).

**Figure 4:**
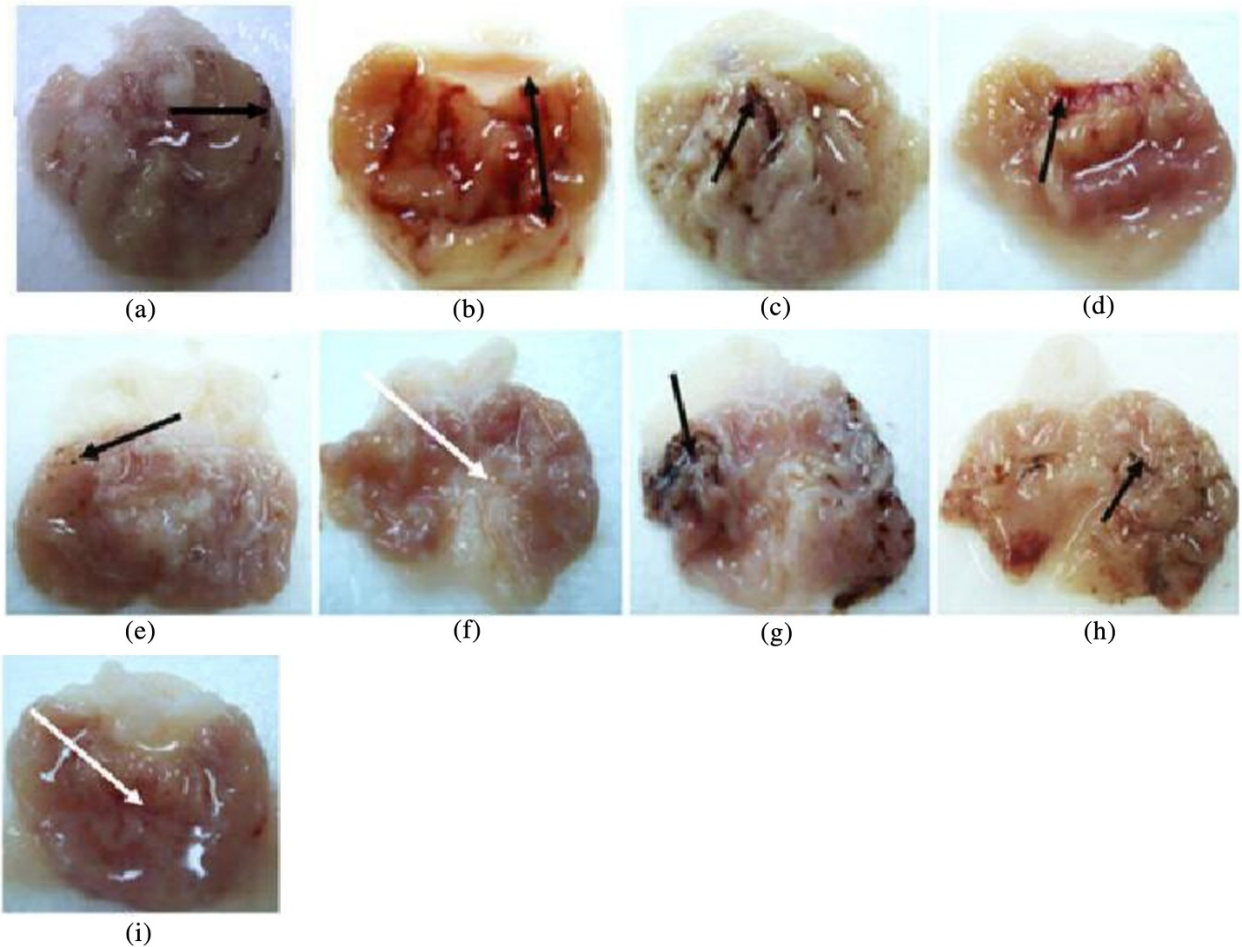
**Gross appearance of the gastric mucosa in rats. (a)** stamach of control (FR). Severe injuries are seen in the gastric mucosa (hemorrhagic necrosis of gastric mucosa). **(b)** stomach of control (MR). Severe injuries are seen in the gastric mucosa (hemorrhagic necrosis of gastric mucosa). **(c)** stomach of rats pre-treated with omeprazole (20 mg/kg). Moderate injuries are seen in the gastric mucosa. **(d)** stomach of female rat pre-treated with EOTa (54 mg/kg). Mild injuries can be seen in the gastric mucosa. **(e)** stomach of female rat pre-treated with EOTa (117 mg/kg). Very mild injuries can be seen in the gastric mucosa. **(f)** stomach of female rat pre-treated with EOTa (800 mg/kg). No injuries can be seen in the gastric mucosa instead flattening of gastric is visible (white arrow). **(g)** stomach of male rat pre-treated with EOTa (54 mg/kg). Severe injuries can be seen in the gastric mucosa. **(h)** stomach of male rat pre-treated with EOTa (117 mg/kg). Severe injuries can be seen in the gastric mucosa. **(i)** stomach of male rat pre-treated with EOTa (180 mg/kg). Very mild injuries can be seen in the gastric mucosa instead flattening of gastric mucosa is visible (white arrow).

The results of antiulcerogenic activity of EOTa on gastric ulcers induced by the HCl/ethanol solution are shown in Figure 4. The acidity of the gastric content in experimental animals pretreated with EOTa (female rats) was decreased significantly compared to that of the ulcer control group (*p < 0.05*). The mucus production of gastric mucosa (Table 3) also increased significantly (*p < 0.05*) in male rats treated with 180 mg/kg and female rats (117 and 180 mg/kg) compared to the ulcer control group.

Oral administration of the damaging agent to the control group clearly produced mucosal damage characterized by multiple hemorrhage red bands of different sizes along the long axis of the glandular stomach as described in other studies [40, 41].

Excessive acidified ethanol ingestion is an etiological factor that gives rise to gastritis characterized by mucosal edema, sub-epithelial hemorrhage, cellular exfoliation and inflammatory cell infiltration [42].

**Figure 5:**
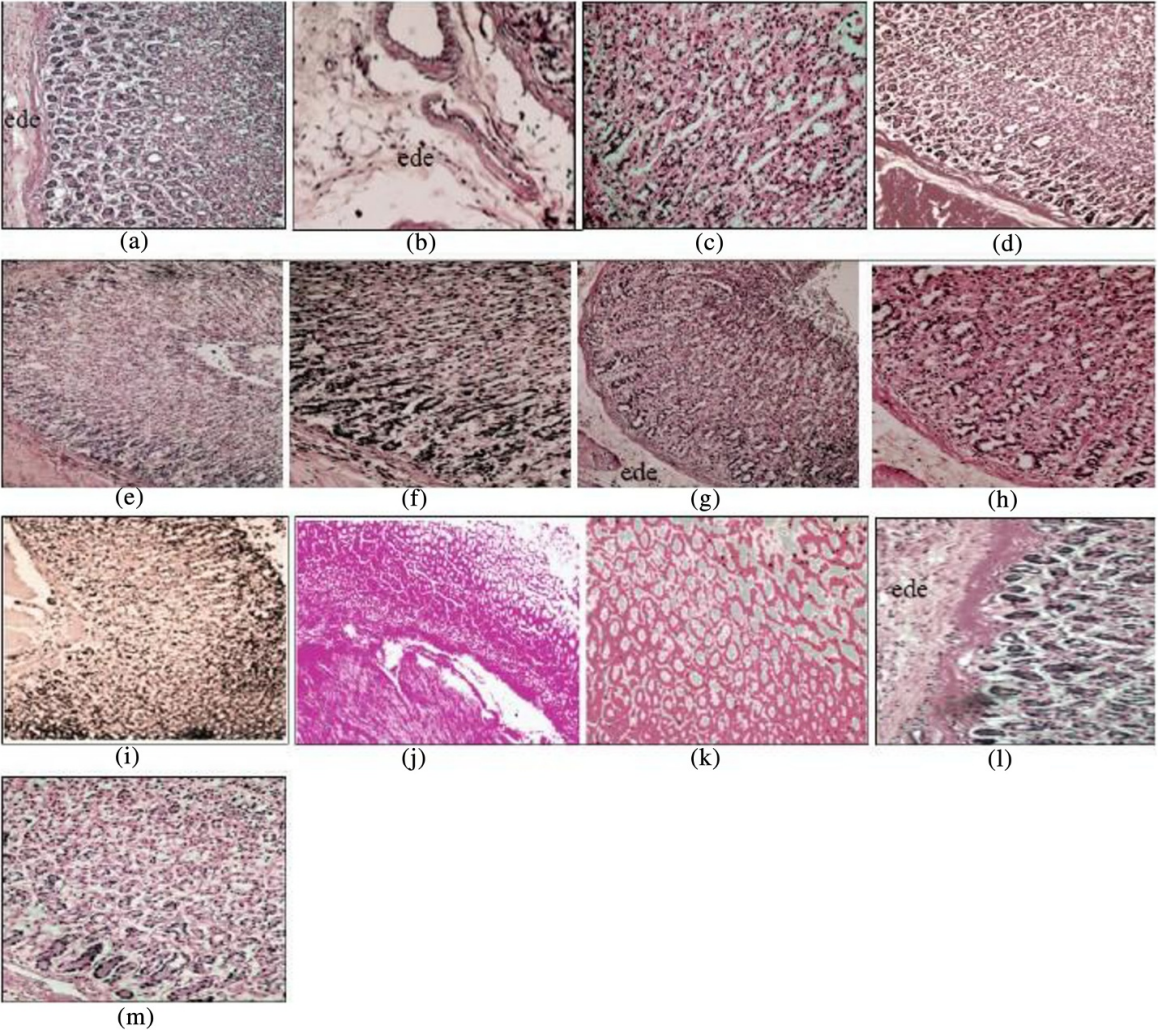
**Photomicrographs of gastric mucosa (Hematoxylin-Eosin staining: magnification 20× for a, b, d, e, g, i, j and l; 40× for c, f, h, k and m). (a)** micrograph of female rats pretreated with HCI/ethanol (ulcer control group), there is epithelial exfoliation and extensive edema (ede) of submucosa layer with distorted glands; **(b, c)** micrograph of male rats pretreated with HCI/ethanol (ulcer control group) shows discontinuity in the lining epithelium with exudates in the lumen submucosal edema (ede) of submucosa layer with distorted glands. **(d)** Omeprazole groups showing epithelium and submucosa with normal characteristics. The EOTa, at 54 mg kg _−1_ **(e, f)**, 117 mg kg-1 **(g,h)** and 180 mg kg_−1_ **(i)** (for FR) showign mocosal regeneration with exudates in lumen with some inflammatory cells, mild mucosal damage, mild leukocyte infiltration, and mild submucosal edema. The EOTa, at 54 mg kg _−1_ **(j, k)** and 117 mg kg _−1_ **(l)** (for MR) showign clearly mucosal hemorrage, submucosal edema, leukocyte and neutrophil infiltration, inflammatory cell and abundant neutrophils, but The EOTa, at 180 mg kg _−1_ **(m)** showing clearly moderate mucosal damage and moderate submucosal edema.

Pretreatment with EOTa (117 ml/kg and 180 ml/kg) significantly reversed these changes for female rats (Figures 4 and 5). Stomachs of male and female rats treated with a higher dose of EOTa showed only mild congestion; otherwise, stomach appearance was normal. There was a clear change in the gross appearance of the gastric mucosa compared to the acidified treated group. Hence, it is likely that the anti-ulceration mechanism of EOTa is due to its antioxidant effect.

In addition, administratering EOTa to female rats was shown to decrease the acidified ethanol-induced gastric ulceration. The mechanism of this protective effect includes the modulatory role of female hormones on vascular permeability and an increase in mucus secretion [43]. In another report, both progesterone and estrogens attenuated the area of acute gastric lesions induced by aspirin and indomethacin [44]. In contrast, testosterone-induced delays in ulcer healing involves the fall in gastric microcirculation at the margin of lingual and gastric ulcers and the excessive production and release of proinflammatory cytokine IL-1b. Treatment with progesterone significantly accelerated ulcer healing and increased the gastric and lingual blood flow at the margin of these ulcers [43].

Our results revealed the protection of the gastric mucosa and inhibition of leukocyte infiltration into the gastric wall in rats pretreated with EOTa. Activation and infiltration of neutrophils appear to be involved in the initial process that forms the lesions [45]. Neutrophil accumulation in gastric mucosa has been shown to induce microcirculatory abnormalities [46]. The present study established that pretreatment with EOTa reduced neutrophil infiltration into ulcerated tissue.

The formation of gastric mucosal lesions by necrotizing agents such as HCl and EtOH has been reported to involve the depletion of gastric defensive mechanisms [47].

Of all these changes, the most prominent are increased capillary permeability and production of free radicals [48], which attack and damage cell membranes, attract neutrophils and initiate an inflammatory response [49].

This was further substantiated by histological findings where a marked reduction in gastric mucosal damage and cellular influx was observed. Some consistent treatment-related histopathological abnormalities were found in rats of either sex. These results indicated that EOTa exhibited a protective effect against HCl/Ethanol-induced ulcerogenesis in rats. Omeprazole, on the other hand, was effective in alleviating oxidative stress in the HCl/Ethanol model. Omeprazole is the first of a new class of drugs that inhibit gastric secretion by altering the activity of H^+^/K^+^-ATPase [50, 51]; it is not charged and can cross cell membranes [52]. Due to being a weak base, omeprazole accumulates in the acidic space of the parietal cell and, by acid-catalyzed rearrangement, becomes a thiol-reactive cationic sulfenic acid and/or sulfenamide that binds to cysteinyl-SH groups to form disulfides [10].

The levels of lipid peroxidation products, GSH and activities of enzymic antioxidants in gastric mucosa of experimental animals are tabulated in Table 4. HCl/ethanol in female rats (180 mg/kg) significantly increased ulceration with a concomitant decrease in LPO levels (0.019 ± 0.09 mmol/mg vs 0.190 ± 0.02 and 0.224 ± 0.02 mmol/mg, P < 0.001) while SOD (178.66 ± 0.01 U/mg protein vs 17 ± 0.045 and 15.38 ± 0.06 U/mg protein, P < 0.001), GSH (3.798 ± 0.23 μmol/mg protein vs 0.085 ± 0.84 0.050 ± 0.00 μmol/mg protein, P < 0.001), GPx (6.1 ± 0.75 μmol GSH/mg protein/ml vs 1.82 ± 0.54 and 1.25 ± 0.09 μmol GSH/mg protein/ml, P < 0.001), GST (9.07 ± 0.72 nmol/min/mg protein vs 0.9 ± 0.67 and 0.82 ± 0.02 nmol/min/mg protein, P < 0.001) and CAT (20.24 ± 0.08 μmol H_2_O_2_ consumed/min/mg protein vs 2.13 ± 0.33 and 2.49 ± 0.87 μmol H_2_O_2_ consumed/min/mg protein, P < 0.001) levels were markedly increased compared to the disease control in gastric mucosal homogenate. In this study SOD, CAT, GPx, GST & GSH activities were significantly elevated by the administration of EOTa to treat rats, suggesting that it has the ability to restore these enzymes.

**Table 4:** **Measurement of the total protein concentration, antioxidant activity, lipid peroxidation of the tissue homogenates**

Groups	Lipid peroxidation nmol MDA/mg protein	Protein concentration (μg/ml)	GSH (μmol/mg protein)	SOD U/mg protein	CAT μmol H_2_O_2_consumed /min/mg protein	GPx μmol GSH/mg protein/ml	GST nmol of CDNB conjugate formed/min/mg protein
Group 1	0.190^*^ ± 0.02	23.75^*^ ± 0.75	0.085^*^ ± 0.84	17^*^ ± 0.45	2.13^*^ ± 0.33	1.82^*^ ± 0.54	0.9^*^ ± 0.67
Group 2	0.224^*^ ± 0.02	16.25^*^ ± 0.08	0.050^*^ ± 0.00	15.38^*^ ± 0.06	2.49^*^ ± 0.87	1.25^*^ ± 0.09	0.82^*^ ± 0.02
Group 3	0.098^#^ ± 0.03	113.75^#^ ± 0.54	1.282^#^ ± 0.51	94.54^#^ ± 0.02	11.15^#^ ± 0.72	4.82^#^ ± 0.68	6.44^#^ ± 0.05
Group 4	0.134^*#^ ± 0.03	47.5^*#^ ± 0.76	0.55^*#^ ± 0.71	23.25^*#^ ± 0.04	8.94^*#^ ± 0.02	1.81^*#^ ± 0.19	0.84^*#^ ± 0.04
Group 5	0.093^*#^ ± 0.02	90^*#^ ± 0.05	1.260^*#^ ± 0.76	27.85^*#^ ± 0.06	4.25^*#^ ± 0.80	2.22^*#^ ± 0.66	1.02^*#^ ± 0.08
Group 6	0.024^*#^ ± 0.03	191^*#^ ± 0.21	3.895^*#^ ± 0.79	91.85^*#^ ± 0.03	19.68^*#^ ± 0.40	4^*#^ ± 0.05	4.96^*#^ ± 0.37
Group 7	0.036^*#^ ± 0.08	122.5^*#^ ± 0.42	1.926^*#^ ± 0.60	121.73^*#^ ± 0.01	11.46^*#^ ± 0.90	3.8^*#^ ± 0.78	6.93^*#^ ± 0.57
Group 8	0.021^*#^ ± 0.01	178.75^*#^ ± 0.18	2.880^*#^ ± 0.12	153.33^*#^ ± 0.07	18.31^*#^ ± 0.10	5.82^*#^ ± 0.98	7.24^*#^ ± 0.73
Group 9	0.019^*#^ ± 0.09	193^*#^ ± 0.02	3.798^*#^ ± 0.23	178.66^*#^ ± 0.01	20.24^*#^ ± 0.08	6.1^*#^ ± 0.75	9.07^*#^ ± 0.72

This experiment consists of the control (FR) (Group 1), the control (MR) (Group 2), the reference group pretreated with 20 mg kg^−1^ of omeprazole (Group 3), the experimental groups MR (Groups 4–6): received 54 mg/kg, 117 mg/kg and 180 mg/kg of the EOT as a pre-treatment and the experimental groups FR (Groups 7–9): received 54 mg/kg, 117 mg/kg and 180 mg/kg of the EOT as a pre-treatment. Value are expressed as mean ± SEM (n = 6), Significant difference at *P* < 0.05 (ANOVA followed by Dunnett’s test) compared with normal control and ulcer control group, MDA, Malondialdehyde; SOD, superoxide dismutase; CAT, catalase; GSH, reduced glutathione; GPx, glutathione peroxidase; GST, glutathione transferase; ^*^significant when compared to the ulcer control groups (1 and 2); ^#^significant when compared to the reference control group (3).

The increase in LPO may suggest a possible mechanism of tissue injury by reactive oxygen intermediates [53]. Hydroxyl radicals thus generated oxidize important cellular constituents such as structural and functional proteins, and membrane lipids as well as deplete glutathione. Lipid peroxidation causes loss of membrane fluidity, impaired ion transport and membrane integrity and finally loss of cellular functions [54].

**Figure 6:**
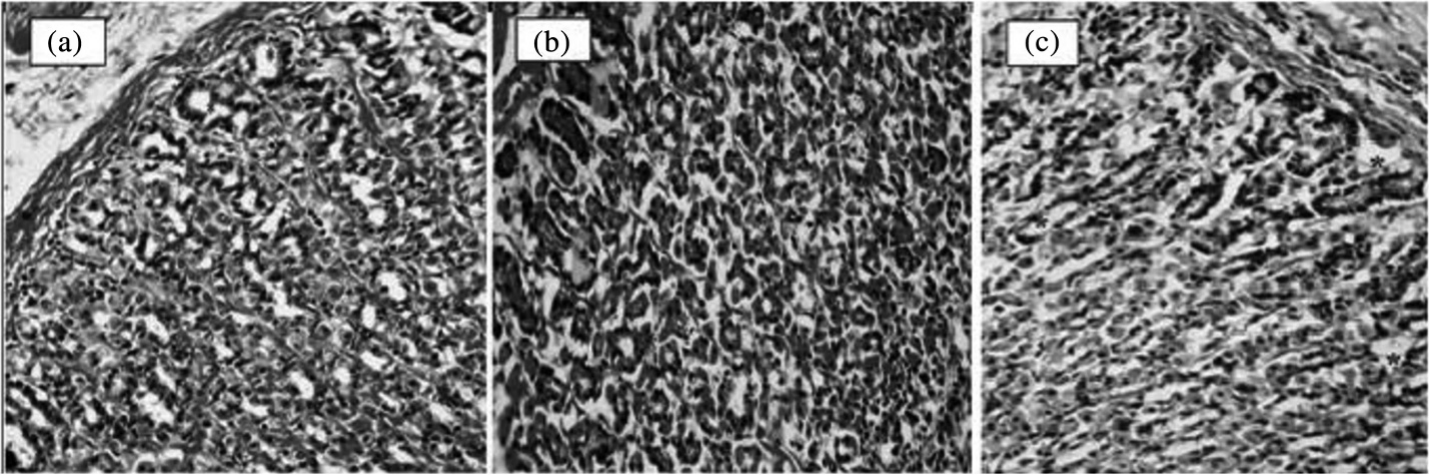
**Photomicrography of histological sections of stomach lesions produced by HCI/ETOH in male and female rats.** Note the secretion inside of glands (*) in treated group with EOTa. Female rat (117 mg/kg) **(a)**, male rat (180 mg/kg) **(b)** and female rat (54 mg/kg) **(c)**. Microscopy magnification 40 ×.

Further, it was observed that in HCl/ethanol- administered rats there was increased generation of reactive oxygen species estimated by increased level of TBARS and attenuated levels of GSH, as well as SOD, CAT and GPx activities and GST along with decreased secretion of mucus (Figure 6) which was reversed in EOTa 180 treatment groups. A significant decrease in gastric GSH following HCl/ethanol administration indicated massive generation of free radicals. Our results corresponded with earlier reports showing a depletion of sulfhydryls in ethanol-induced gastric lesions [55]. Treatment with EOTa produced a significant increase in the level of GSH and activities of SOD, CAT, GPx and GST, and a significant decrease in the level of TBARS in the HCl/ethanol model (Table 4). Reduced glutathione is one of the most abundant non-enzymatic antioxidant bio-molecules present in the tissues [56]. Its functions are removing free oxygen species such as H_2_O_2_, superoxide anions and alkoxy radicals, maintaining membrane protein thiols and acting as a substrate for GPx and glutathione *S*- transferase (GST) [57]. The non-availability of GSH decreases the activities of GSH- dependent enzymes GPx and GST and/or renders these enzymes inactive and/or less active [58].

## Conclusion

It may be concluded that a single oral administration of essential oil from *Thymys algeriensis* possesses a significant gastroprotective effect as assessed by significant antioxidant activity as it attenuated the level of TBARS and elevated levels of GSH, GST, GPx, CAT and SOD. This effect could be related to an increase of gastric mucosal defensive mechanisms. The effectiveness of the essential oil and its low toxicity requires further study to elucidate the action mechanism as well as to isolate the gastroprotective principles. In addition, the histopathological results of our study revealed that treatment with *EOTa* (117 and 180 mg/kg) resulted in maintaining the mucosal integrity and a mild mucosal ulceration.
